# Metagenomic Insights into Effects of Thiamine Supplementation on Carbohydrate-Active Enzymes’ Profile in Dairy Cows Fed High-Concentrate Diets

**DOI:** 10.3390/ani10020304

**Published:** 2020-02-14

**Authors:** Yiguang Zhao, Fuguang Xue, Dengke Hua, Yue Wang, Xiaohua Pan, Xuemei Nan, Fuyu Sun, Linshu Jiang, Benhai Xiong

**Affiliations:** 1 State Key Laboratory of Animal Nutrition, Institute of Animal Sciences, Chinese Academy of Agricultural Sciences, Beijing 100193 China; zhaoyiguang@caas.cn (Y.Z.); xuefuguang123@163.com (F.X.); dengke_h@163.com (D.H.);; 2 College of Animal Science and Technology, Jiangxi Agricultural University, Nanchang 330045, China; 3 Beijing Key Laboratory for Dairy Cow Nutrition, Beijing University of Agriculture, Beijing 102206, China

**Keywords:** carbohydrate-active enzymes, dairy cows, metagenome, thiamine

## Abstract

**Simple Summary:**

Overfeeding a high-grain diet is known to reduce ruminal pH and microbial activity, disrupt carbohydrate metabolism, and consequently lead to subacute ruminal acidosis. Thiamine, as the co-enzyme of pyruvate formate-lyase, plays a critical role in carbohydrate metabolism in dairy cows. Therefore, we investigated the impacts of thiamine supplementation on ruminal carbohydrate-active enzymes using the metagenomic sequencing technique in lactating dairy cows fed high-concentrate diets. The results indicated that feeding high concentrate diets reduced fiber-degrading enzymes and the total carbohydrate-active enzymes. However, dietary thiamine supplementation increased fiber-degrading enzymes, starch-degrading enzymes, and total carbohydrate-active enzymes. These findings demonstrated that thiamine supplementation could enhance rumen carbohydrate metabolism through increasing the abundance of ruminal carbohydrate-active enzymes, providing effective strategies to improve dairy cows’ health and wellbeing under a high-concentrate feeding regime.

**Abstract:**

As the co-enzyme of pyruvate formate-lyase under ruminal anaerobic condition, thiamine plays a critical role in carbohydrate metabolism in dairy cows. The objective of this study was to investigate the impacts of thiamine supplementation on ruminal carbohydrate-active enzymes. Twelve Holstein dairy cows were randomly assigned into three dietary treatments: control diet (CON; 20% starch, dry matter (DM) basis), high-concentrate diet (HC; 33.2% starch, DM basis) and a high-concentrate diet supplemented with 180 mg thiamine/kg DM (HCT; 33.2% starch, DM basis). Dry matter intake and milk production were recorded for 21 days. Rumen fluid samples were collected, and ruminal pH and volatile fatty acids (VFAs) were measured. The metagenome sequencing technique was used to detect the genes in ruminal microorganisms and identify putative carbohydrate-active enzymes. The total abundances of carbohydrate-active enzymes and fiber-degrading enzymes were both reduced by HC with no effect on starch-degrading enzymes compared with CON. However, the fiber-degrading enzymes and starch-degrading enzymes were both increased after thiamine supplementation. These results indicated that 180 mg thiamine /kg DM might effectively improve rumen carbohydrate metabolism through increasing the abundance of ruminal carbohydrate-active enzymes and consequently balanced the rumen volatile fatty acids and rumen pH, providing a practical strategy in preventing subacute ruminal acidosis in cows offered HC.

## 1. Introduction

Carbohydrates, usually making up 70–80% of the diets, are the primary energy sources for the high requirement of lactating dairy cows [1,2]. The rumen microbiota provides series of enzymes for degradation of carbohydrates and, thereafter supply essential substrates (i.e., volatile fatty acids (VFAs) and microbial protein) for the growth and lactation of dairy cows [3,4]. Typically, carbohydrates contain cellulose, hemicellulose, and starch, which are derived from forage and grain of the diet. In ruminal condition, cellulose and starch are digested by cellulase and amylase secreted by rumen microbiota into disaccharide, which is then degraded into glucose [5,6]. Appropriate dietary ratio of forage to grain could optimize the fermentation condition for ruminal microbiota and provide balanced energy and nutrients for the animals [7,8]. However, in modern dairy cattle production systems, overfeeding of high-concentrate diet (HC), mostly rich in starch, frequently leads to the destabilization of the ruminal microbial ecosystem [4,9] and consequently disrupted carbohydrate metabolism [10,11]. Therefore, developing a proper strategy to alleviate HC induced metabolic disorders is of great importance in high-yielding dairy cattle production systems.

Our previous studies found that thiamine supplementation could effectively attenuate subacute ruminal acidosis (SARA) induced by a high-grain diet through buffering ruminal pH, shifting rumen fermentation pattern, and balancing the structure of ruminal microorganisms [12,13]. Thiamine (C_12_H_16_N_4_OS), also known as vitamin B1, plays a critical role in carbohydrate metabolism [14] and has been proved to be the co-enzyme of pyruvate formate-lyase (PFL) which promotes pyruvate hydrolyzing into acetyl-CoA and reduces the accumulation of lactate [13]. The promoted reaction of pyruvate to acetyl-CoA may benefit ruminal carbohydrate metabolism. Since carbohydrate metabolism is catalyzed by carbohydrate-active enzymes (CAZymes), a hypothesis was made that thiamine supplementation might increase the CAZymes profile to promote ruminal carbohydrate metabolism.

The CAZymes database (http://www.cazy.org/) is the key database that describes the families of structurally-related catalytic and carbohydrate-binding modules (or functional domains) of enzymes that degrade, modify, or create glycosidic bonds [15] and has been widely applied in ruminal enzymatic researches [16,17]. The CAZymes database currently contains glycoside hydrolases (GHs), glycosyl transferases (GTs), polysaccharide lyases (PLs), carbohydrate esterases (CEs), carbohydrate-binding modules (CBMs), and auxiliary activities (AAs). Additionally, in combination with the metagenome technology, which provides enormous functional screening of clones of uncultured microbes and valuable alternatives for identifying putative CAZymes, it could contribute to better understanding the ruminal carbohydrate metabolism [18,19]. Therefore, metagenomic sequencing was applied in the present study to align the CAZymes database to investigate the effects of thiamine supplementation on the ruminal CAZymes profile and the carbohydrate metabolism in HC feeding dairy cows.

## 2. Materials and Methods

### 2.1. Animal Preparation

Animals were selected from the dairy cattle farm of the Institute of Animal Science, Chinese Academy of Agricultural Sciences, Beijing, China. Animal care and procedures were in accordance with the Chinese guidelines for animal welfare and approved by the Animal Care and Use Committee of the Chinese Academy of Agricultural Sciences (IAS2019-55). Throughout the experimental period, the cows were housed in a single dairy barn area (18 m long × 4.25 m wide × 4.5 m high) with free access to fresh water.

### 2.2. Experimental Design and Dietary Treatments

The experimental design has been stated in our previous study [20]. In brief, twelve Holstein dairy cows (627 ± 19.9 kg BW (body weight); 180 ± 8 DIM (days in milk)) in second-parity fitted with ruminal cannulas (φ = 10 cm, Bar Diamond, Parma, ID) were randomly assigned into three treatments: a control diet (CON; 20% starch, dry matter (DM) basis), a high-concentrate diet (HC; 33.2% starch, DM basis), and high-concentrate diet supplemented with 180 mg thiamine/kg DM (HCT, 33.2% starch, DM basis). A previous study found that adding 180 mg thiamine/kg DM in HC could increase the rumen pH and regulate the structure of rumen microbial community in vivo [21]. Thiamine (thiamine hydrochloride, purity ≥ 99%; Wanrong Science and Technology Development Co. Ltd., Wuhan, China) was added through the rumen cannula. Ingredients and chemical composition of the diets are shown in Supplementary Table S1. Cows were fed twice equally at 6:00 and 18:00 each day ad libitum and were milked three times a day in the morning, midday, and evening for 21 days.

### 2.3. Rumen Fluid Sampling and Parameter Measurement

During the experimental period, automatic feeding equipment (Institute of Animal Science, Chinese Academy of Agricultural Sciences, Beijing, China, and NanShang Husbandry Science and Technology Co. Ltd., Zhengzhou, China) was used to record dry matter intake (DMI). Milking facilities of Afimilk (Side-by-Side Parallel Stall Construction, Afimilk Ltd., Kibbutz Afikim, Israel) were applied to record milk production of each cow. Two hundred and fifty milliliters of rumen semi-solid contents of each cow were sampled at 3 h after the morning feeding on day 21. Rumen contents were strained through 4 layers of cheesecloth with a mesh size of 250 μm. Rumen fluid was then divided into two portions. One was used to analyze the pH value, thiamine, and rumen volatile fatty acid (VFA) concentrations. Ruminal pH was measured immediately using a portable type pH meter (Testo 205, Testo AG, Lenzkirch, Germany). Then the rumen fluid was centrifuged at 10,000× *g* for 15 min at 4 °C. Five mL of clear supernatant samples was transferred into tubes and stored at −20 °C for analysis of thiamine. Another 10 mL of clear supernatant samples was mixed with 2 mL of 250 g/L of metaphosphoric acid and stored at −20 °C for VFA analysis. The concentrations of VFA and thiamine were determined, as described by Pan et al. [22]. The other portion was frozen in the liquid nitrogen immediately after adding stabilizer and then stored at −80 °C for DNA extraction.

### 2.4. Metagenomic Sequencing Analysis

Metagenomic sequencing analysis of DNA extraction, library construction, sequence quality control, genome assembly, gene prediction, and taxonomy was performed as described in our previous study [20]. In general, DNA was extracted from the rumen fluid samples using the QIAamp DNA Stool Mini Kit (Qiagen, Hilden, Germany). The DNA concentration and purity were quantified by a TBS-380 (P/N, 3800-003, Turner Biosystem, Sunnyvale, CA, USA) and a NanoDrop 2000 (Thermo Fisher Scientific, Waltham, MA, USA), respectively. DNA was fragmented to an average size of 300 bp using Covaris M220 (Gene Company Limited, Hong Kong, China) for paired-end library construction, and the library was prepared using a TruSeq DNA Sample Prep Kit (Illumina Inc., San Diego, CA, USA). Paired-end sequencing was performed on an Illumina HiSeq 4000 platform (Illumina Inc., San Diego, CA, USA) using a HiSeq 3000/4000 PE Cluster Kit (www.illumina.com, Illumina Inc., San Diego, CA, USA).

The quality control methods of reads were stripped using SeqPrep (https://github.com/jstjohn/SeqPrep). The low-quality reads, or reads associated with the cow genome, were removed [23]. The filtered reads were used in assembly by SOAP de novo (http://soap.genomics.org.cn, Version 1.06) based on De Brujin graph construction [24]. Scaffolds with a length over 500 bp were retained and then extracted and broken into contigs without gaps. Contigs were used for further gene prediction and annotation.

Open reading frames (ORFs) from each sample were predicted using MetaGene (http://metagene.cb.k.u-tokyo.ac.jp/) [25]. DIAMOND [26] was employed for taxonomic annotations by aligning non-redundant gene catalogs against the National Center for Biotechnology Information (NCBI) non-redundant protein sequence database with an e-value cutoff of 1 × 10^−5^ and Score > 60. Based on the NCBI microbial taxonomy information database, species annotation information of genes was obtained, and the relative abundance of species was calculated.

GhostKOALA (https://www.kegg.jp/ghostkoala/) [27] was employed for the KEGG pathway annotation against the Kyoto Encyclopedia of Genes and Genomes database (http://www.genome.jp/kegg/) with an e-value cutoff of 1 × 10^−5^. Annotate sequences in the KEGG Mapper were specially interfaced with the BlastKOALA server and executed in an interactive mode. CAZymes annotations were then conducted using hmmscan (http://hmmer.janelia.org/search/hmmscan) to match the CAZymes database V5.0 (http://www.cazy.org/). The results were considered significantly enriched in CAZymes with an e-value cutoff of 1 × 10^−5^.

Nucleotide Sequence Accession Number.

All the raw sequences were submitted to the NCBI Sequence Read Archive (SRA; http://www.ncbi.nlm.nih.gov/Traces/sra/) under accession number SRP144478.

### 2.5. Statistical Analysis

Effects of high-concentrate diet and thiamine supplementation treatment on the relative abundances of genes encoding carbohydrate-active enzymes, fiber-degrading enzymes, and starch-degrading enzymes were analyzed using the Student–Newman–Keuls (SNK) test of SAS 9.22 (SAS Institute Inc., Cary, NC, USA). *p* < 0.05 was considered as significant and 0.05 ≤ *p* < 0.10 was considered as a tendency. Correlation distance analysis of CAZymes was conducted among the three dietary treatments using the R package (version 3.3.1; https://www.r-project.org/). Correlation analysis between DMI, milk fat, milk protein, ruminal fermentation parameters (pH, thiamine, acetate, propionate, butyrate, valerate, and isovalerate concentrations) and relative abundances of fiber-degrading enzymes and starch-degrading enzymes, respectively, were assessed using the PROC CORR procedure of SAS 9.22 (SAS Institute Inc., Cary, NC, USA). A correlation matrix was then created and visualized in a heatmap format using the R package (version 3.3.1; https://www.r-project.org/). The relationships were considered significant with the absolute value of correlation coefficients (|r|) > 0.55 and *p* < 0.05.

## 3. Results

### 3.1. Animal Performance and Rumen Fermentation Parameters

The DMI, milk production, ruminal pH, and ruminal VFAs parameters have been stated in our previous study [20], which are also provided in Supplementary Table S2. In brief, the results indicated that HC feeding significantly decreased DMI, milk production, milk fat, ruminal pH, thiamine, and acetate concentrations; however, it significantly increased propionate, valerate, and isovalerate concentrations compared with CON treatment (*p* < 0.05). In contrast, these changes were significantly inversed by thiamine supplementation (*p* < 0.05). While butyrate and total VFA concentrations were not affected by dietary treatments (*p* > 0.05).

### 3.2. Sequencing Quality

The sequencing results are shown in Supplementary Table S3. Twelve metagenomic libraries were constructed in the current study. Approximately 45,000,000 reads, and a minimum of 130,000 contigs of each sample were obtained. The contigs were then aligned to the NCBI NR database to identify the predicted genes, and more than 200,000 predicted genes were identified in each sample, which were then used for functional analysis.

The KEGG database was used to annotate the genes that might participate in ruminal metabolism through MetaGene (http://metagene.cb.k.utokyo.ac.jp/) [15]. More than 70% of the predicted genes were enriched in nutrient metabolism pathways, most of which were enriched in carbohydrate pathways. Based on the KEGG results, the identified genes were aligned to the CAZymes database, and 4.65–6.80% of the genes were matched to CAZymes database V5.0, in which 224 CAZymes were identified. Among these enzymes, 10 AAs, 33 CBMs, 16 CEs, 97 GHs, 54 GTs, and 15 PLs were identified, respectively. These results are shown in Supplementary Excel S1.

### 3.3. Effects of Thiamine Supplementation on CAZymes

Differential analysis of all CAZymes categories was then conducted to investigate the effects of HC and thiamine supplementation on CAZymes. The results are shown in Table 1. The total genes matching the CAZymes database in the HC treatment was significantly less than those in CON, which were, however, significantly increased after thiamine supplementation (*p* < 0.001), while there was no significant difference between CON and HCT. In detail, genes encoding CBMs, CEs, GHs, GTs, and PLs were all less abundant in HC than those in CON, which were then significantly increased after thiamine supplementation in comparison with HC (*p* < 0.01). The abundances of genes that encoded AAs and GTs in HCT were higher than both of CON and HC (*p* < 0.001).

**Figure 1:**
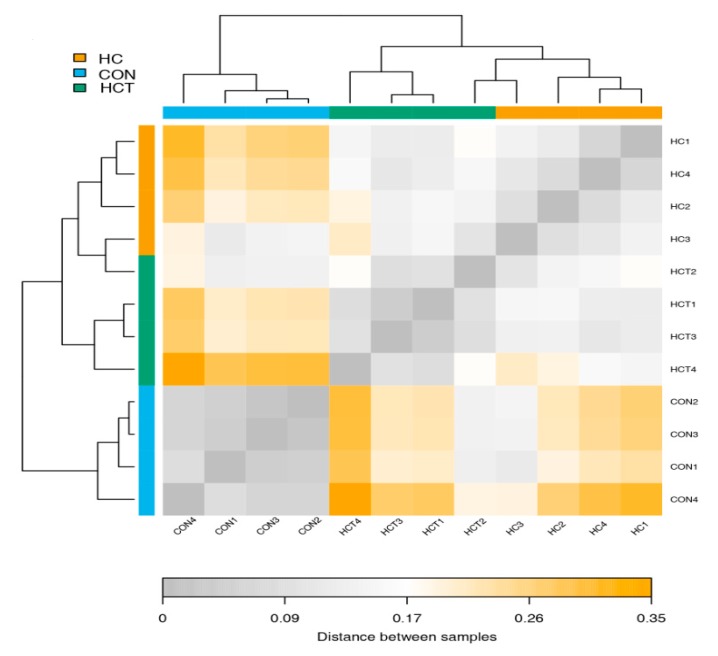
Correlation-distance analysis of carbohydrate-active enzymes (CAZymes) among CON, HC, and HCT treatments. The darker of the block color means the closer distance between samples. CON = control diet, HC = high-concentrate diet, HCT = high-concentrate diet supplemented with 180 mg thiamine/kg dry matter (DM).

The correlations of CAZymes of each sample among the three treatments were investigated using correlation-distance analysis (Figure 1). The results indicated that CAZymes of CON gathered into a big cluster and were separated from those of HC and HCT. Meanwhile, CAZymes of HCT could also be separated from those of HC except HCT 2.

### 3.4. Effects of Thiamine Supplementation on Fiber-Degrading Enzymes and Starch-Degrading Enzymes

Differential analysis was then used to investigate the effects of the dietary treatments on the relative abundance of genes encoding fiber-degrading enzymes and starch-degrading enzymes based on the functional annotation of CAZymes database (http://www.cazy.org/) according to the report of Comtet-Marre et al. [15] in Table 2 and Table 3, respectively. HC significantly decreased the total relative abundances of fiber-degrading enzymes compared with CON (*p* < 0.001). In contrast, thiamine supplementation significantly increased the total relative abundances of fiber-degrading enzymes compared with both HC and CON (*p* < 0.001). Specifically, HC significantly reduced the abundances of genes encoding CBM3, CBM30, GH11, and GH48 compared with CON (*p* < 0.01), and HCT significantly increased the abundances of genes encoding GH10, GH45, and GH5 in comparison with CON and HC (*p* < 0.01). In which, GH10 and GH45 genes were the main genes that contributed to the significant effect on the higher total abundance of genes encoding fiber-degrading enzymes in HCT than CON and HC.

On the other hand, HCT significantly increased the total relative abundances of starch-degrading enzymes compared with CON and HC (*p* = 0.016), while no difference was detected between the latter two. For individual enzymes, HC had significantly fewer abundances of genes encoding CBM20, GH13, GH4, GH63, and greater GH97 than CON (*p* < 0.05). Meanwhile, CBM25, GH15, and GH119 genes were all most abundant in HCT in comparison with CON and HC (*p* < 0.01), which contributed to the significant effect on the higher total abundance of genes encoding starch-degrading enzymes in HCT than CON and HC.

### 3.5. Relationships between CAZymes and Animal Performance

**Figure 2:**
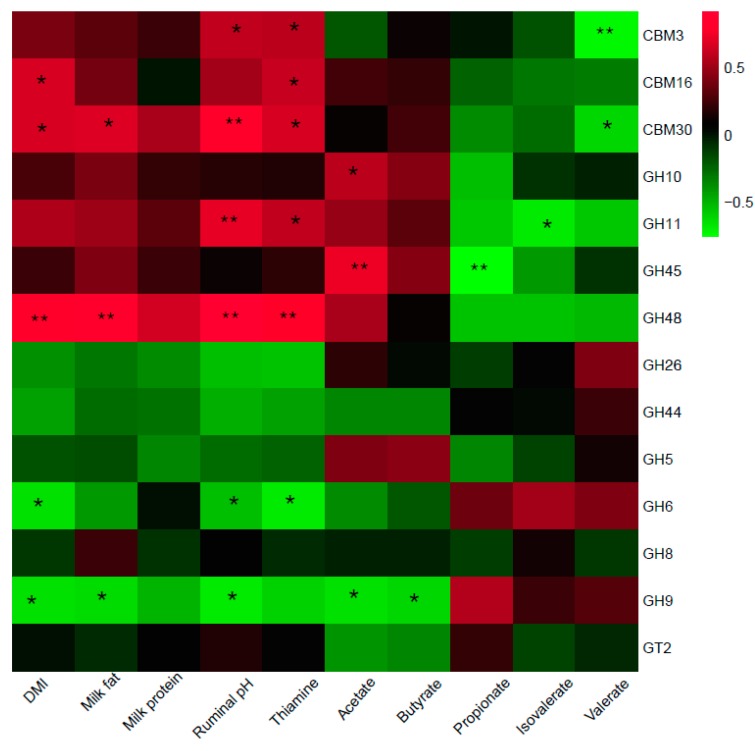
Correlation analysis between relative abundances of fiber-degrading enzymes and dry matter intake (DMI), milk fat, milk protein, and ruminal fermentation parameters. CBM = Carbohydrate-binding modules, GH = Glycoside hydrolase, GT = Glycosyl transferase. Red blocks represent positive correlations. Green blocks represent negative correlations. “*” and “**” mean significant correlations with |r| > 0.55 and *p* < 0.05, *p* < 0.01, respectively.

Finally, a Spearman correlation analysis between CAZymes and animal performance (DMI, milk quality, and ruminal fermentation parameters) was applied. The results of the correlation analysis between fiber-degrading enzymes and animal performance are shown in Figure 2. In which, CBM3 was positively correlated with ruminal pH, thiamine, and negatively correlated with valerate. CBM16 was positively correlated with DMI and thiamine. CBM30 had positive correlations with DMI, milk fat, ruminal pH, and thiamine and a negative correlation with valerate. For GHs, GH10 and GH45 both had positive relationships with acetate, while GH45 had a negative relationship with propionate. GH11 was positively correlated with ruminal pH and thiamine and negatively correlated with isovalerate. GH48 was positively correlated with DMI, milk fat, ruminal pH, thiamine. While GH6 was negatively correlated with DMI, ruminal pH, thiamine. In addition, GH9 had negative relationships with DMI, milk fat, ruminal pH, acetate, and butyrate.

**Figure 3:**
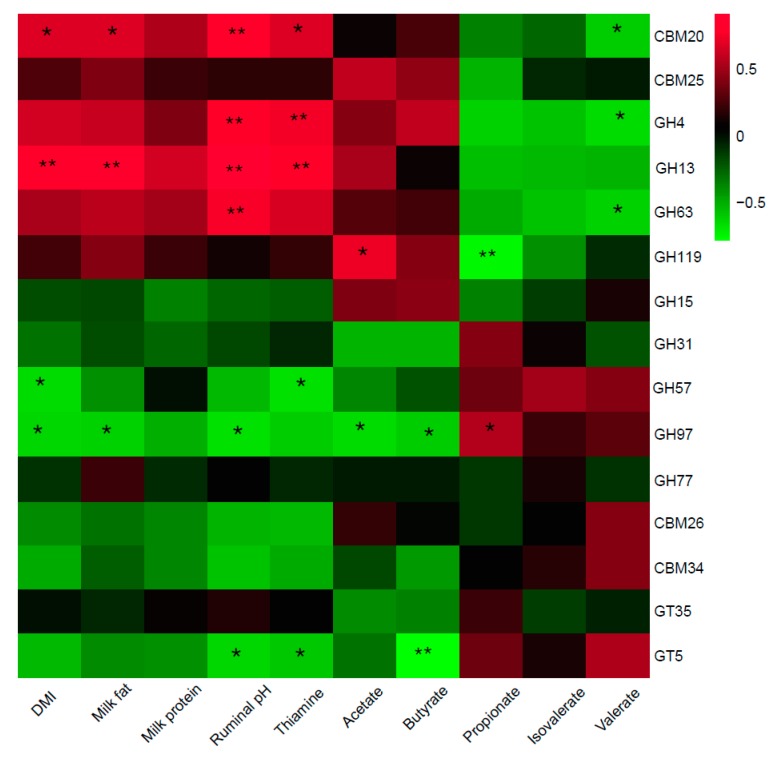
Correlation analysis between relative abundances of starch-degrading enzymes and DMI, milk production, milk quality, and ruminal fermentation parameters. CBM = Carbohydrate-binding modules, GH = Glycoside hydrolase, GT = Glycosyl transferase. Red blocks represent positive correlations. Green blocks represent negative correlations. “*” and “**” mean significant correlations with |r| > 0.55 and *p* < 0.05, *p* < 0.01, respectively.

Similarly, the results of correlation analysis between starch-degrading enzymes and animal performance are shown in Figure 3. Positive correlations were detected between CBM20 and DMI, milk fat, and rumen pH and thiamine concentration, while there was a negative relationship between CBM20 and rumen valerate content. For GHs, GH4 and GH63 were both positively correlated with rumen pH and negatively correlated with valerate, while GH4 was also positively correlated with rumen thiamine. GH13 was found positively correlated with DMI, milk fat, rumen pH, and thiamine. GH119 had a positive relationship with acetate and a negative relationship with propionate. In contrast, GH97 had negative relationships with acetate, butyrate, rumen pH, milk fat, and DMI, and a positive relationship with propionate. Furthermore, GH57 was negatively correlated with DMI and rumen thiamine. Negative relationships were also detected between GT5 and rumen pH, thiamine, and butyrate.

## 4. Discussion

### 4.1. Effects of Thiamine Supplementation on Fiber-Degrading Enzymes

Ruminants have a special capacity to digest fibers in the feeds with the assistance of ruminal microorganisms [28]. Approximately 35–55% of cellulose and hemicellulose in the diet could be degraded by the rumen microbes [29]. In the present study, thiamine supplementation significantly increased the relative abundances of genes encoding CAZymes that mainly degraded cellulose and hemicellulose by 52.8% compared with HC. Previous studies of our team discovered that thiamine supplementation enriched the ruminal microbiome [12], especially the ruminal fungi [20]. All fungal species were found to express an extensive array of transcripts encoding CAZymes ranging from 8.3% to 11.3% of the transcriptome [30]. Therefore, the improved abundance of fungi may contribute to the increased abundance of fiber-degrading enzymes-encoding genes in the HCT in the present study. In anaerobic fungi, CAZymes can be found as free enzymes or in a cellulosome [29]. The extensive CAZymes profile and cellulosome in anaerobic fungi may explain their strong fiber degrading ability. Besides, the genome sequencing of anaerobic fungi suggests that many fungal CAZymes could be acquired by horizontal gene transfer from rumen bacteria [31,32]. The interaction between rumen fungi and bacteria might further promote the fiber-degrading CAZymes’ profile and consequently improve fiber digestion.

In addition, fiber mainly contains cellulose and hemicellulose. Where cellulose is composed of glucose and hemicellulose is composed of linear and branched heteropolymers of o-xylose, L-arabinose, D-mannose, D-glucose, D-galactose, and D-glucuronic acid [33]. In ruminal condition, thiamine has been proved to be the cofactor of pyruvate formate-lyase (PFL) catalyzing pyruvate into acetyl-CoA and thereby facilitates the hydrolysis of monosaccharide [20]. The promoted degrading of monosaccharide may improve the digestion of cellulose and hemicellulose through positive feedback and provide more energy to simulate microbes to generate more fiber-degrading CAZymes.

Acetate is produced from acetyl-coA [34], while thiamine supplementation promoted pyruvate into acetyl-coA instead of oxaloacetic acid, providing more substrates for the synthesis of acetate rather than propionate [35]. This could possibly explain why HCT increased rumen acetate concentration compared with HC. Meanwhile, thiamine supplementation also increased the total profile of fiber-degrading enzymes and many individual enzymes, such as GH5 and GH45 (the main cellulose-degrading enzyme family) [36,37] and GH10 (the main hemicellulose-degrading family) [38]. Therefore, it is reasonable that GH10 and GH45 were positively correlated with rumen acetate concentration in the present study.

### 4.2. Effects of Thiamine Supplementation on Starch-Degrading Enzymes

Similar to cellulose and hemicellulose, more than 60% of starch is digested by the rumen microbes, varying according to the feed intake and its passage rate through the rumen [39]. In the present study, the total relative abundances of genes encoding starch-degrading enzymes were not affected by HC, however, were significantly increased by 46.7% after thiamine supplementation compared with HC. These results indicated that thiamine supplementation had the effect of promoting starch-degrading enzymes. The optimum pH of starch-degrading enzymes varied from 2.0 to 10.5, indicating their extensive adaptability to the ruminal environment [39]. This might explain the stability of starch-degrading enzymes in the HC feeding treatment.

Dietary starch is hydrolyzed into glucose and then digested into pyruvate and acetyl-CoA by microorganisms through the glycolytic pathway and pentose phosphate pathway [34,40]. However, because of the rapid fermentation of starch and the limited glucose decomposition capability, glucose is often accumulated in the rumen with high concentrate diets. It has been reported that the accumulation of glucose caused repression of amylase synthesis of ruminal microbiota, such as *Streptococcus bovis* [41] and *Clostridium acetobutylicum* [42]. This might explain why starch-degrading enzymes were not increased in HC feeding treatment. In contrast, as stated previously, thiamine supplementation catalyzed pyruvate into acetyl-CoA, indirectly leading to the increased hydrolysis of glucose, which, therefore, eliminated the repression of glucose on ruminal microbiota, and consequently increased the starch-degrading enzymes profile.

To effectively degrade ruminal starch, many microbial GHs are appended with CBMs [43]. The CBMs convert carbohydrates into structure favoring substances and provide noncatalytic modules with enhanced ability to bind onto specific polysaccharide surfaces for subsequent enzymatic catalysis [44,45]. CBMs that enhance the binding ability and degradation of starch are mainly present in GH13 and GH15 [46], which were identified in the present study as the two most abundant starch-degrading enzymes and significantly increased by thiamine supplementation. The increased abundances of starch-binding CBMs (e.g., CBM25 and CBM26) after thiamine supplementation may also enhance the ability of starch degradation.

Usually, a high proportion of dietary starch results in the accumulation of propionate in the rumen [47,48]. However, HCT had lower propionate concentration than that in HC and CON in the present study. Propionate is produced through two major routes, including indirectly from lactate via dehydration to acrylate and then reduction reaction to propionate and direct decarboxylation of succinate [34]. It has been reported that thiamine supplementation in HC stimulated the growth of the lactate utilizing bacteria *Megasphaera elsdenii* and reduced the lactate producing bacteria *Strepococcus bovis* and *Lactobacillus* [21]. Furthermore, as the essential cofactor of pyruvate decarboxylation, thiamine improved the decarboxylation of pyruvate to produce acetate, thus reduced the production of succinate [12]. Therefore, the precursors of propionate in both of the direct and indirect pathways, lactate and succinate, were possibly reduced after thiamine supplementation, which might consequently result in the decrease in propionate in HCT. Meanwhile, HCT decreased GH97 and increased GH119, which were thus detected positively and negatively correlated with rumen propionate concentration, respectively, in the current study.

Previous studies of our team proved that 180 mg thiamine/kg DM in HC could help attenuate SARA by increasing ruminal pH, acetate, and thiamine contents and reducing propionate concentration in rumen fluid compared with HC [12,22]. This is also confirmed in the current study when HC and HCT are compared. For example, the ruminal pH was increased from 5.58 to 6.12 and the increased acetate and decreased propionate concentrations by thiamine supplementation as discussed above. The results again indicated that 180 mg thiamine/kg DM was practical in preventing SARA in dairy cows offered HC.

## 5. Conclusions

In the present study, we observed that 180 mg/kg DM of thiamine supplementation in HC significantly increased the relative abundances of the genes encoding CAZymes in dairy cow rumen microorganisms by 33.6% compared with HC, including both fiber (52.8% increase) and starch (46.7% increase) degrading enzymes. These results provided genetic evidences to explain how thiamine supplementation promoted the carbohydrate metabolism in the rumen through metagenomic sequencing of the rumen microorganisms and consequently balanced the rumen VFA and rumen pH, which could thus be a practical strategy in preventing SARA in cows offered HC. The findings added some novel information on thiamine’s function in dairy cows under a high-concentrate feeding regime. However, further research is still needed to investigate the relationships between CAZymes and rumen microbes and the mechanism of correlations between CAZymes and rumen fermentation variables.

## Figures and Tables

**Table 1:** Effects of high-concentrate diet and thiamine supplementation treatment on the relative abundances of genes encoding carbohydrate-active enzymes.

Items	CON	HC	HCT	SEM	*p*-Value
AAs	0.04 ^b^	0.04 ^b^	0.10 ^a^	0.006	<0.001
CBMs	0.34 ^a^	0.26 ^b^	0.35 ^a^	0.029	0.002
CEs	1.36 ^a^	0.97 ^b^	1.33 ^a^	0.117	<0.001
GHs	4.75 ^a^	3.74 ^b^	4.79 ^a^	0.377	<0.001
GTs	1.74 ^b^	1.43 ^c^	2.10 ^a^	0.102	<0.001
PLs	0.30 ^a^	0.24 ^b^	0.31 ^a^	0.025	0.002
Total	8.53 ^a^	6.69 ^b^	8.94 ^a^	0.640	<0.001

^a–c^ mean within a row with different letters differed significantly (*p* < 0.05), SEM = standard error of the mean. CON = control diet, HC = high-concentrate diet, HCT = high-concentrate diet supplemented with 180 mg thiamine/kg DM, AAs = Auxiliary activities, CBMs = Carbohydrate-binding modules, CEs = Carbohydrate esterases, GHs = Glycoside hydrolases, GTs = Glycosyl transferases, PLs = Polysaccharide lyases.

**Table 2:** Effects of high-concentrate diet and thiamine supplementation treatment on relative abundance of genes that encoded fiber-degrading enzymes.

Enzyme	Function	CON	HC	HCT	SEM	*P*-Value
CBM3	cellulose-binding function	9.13 × 10^−3 a^	5.46 × 10^−3 b^	5.45 × 10^−3 b^	6.00 × 10^−4^	0.001
CBM16	binding to cellulose	8.49 × 10^−5^	2.91 × 10^−5^	4.47 × 10^−5^	1.00 × 10^−5^	0.228
CBM30	binding to cellulose	3.43 × 10^−3 a^	1.90 × 10^−3 b^	2.43 × 10^−3 b^	2.30 × 10^−4^	0.008
GH10	cellulase family F.	9.03 × 10^−3 b^	8.30 × 10^−3 b^	1.49 × 10^−2 a^	1.06 × 10^−3^	0.004
GH11	cellulase family G	3.16 × 10^−2 a^	1.82 × 10^−2 b^	2.86 × 10^−2 a^	2.02 × 10^−3^	0.002
GH26	cellulase family I.	1.38 × 10^−3 b^	2.38 × 10^−3 a,b^	3.07 × 10^−3 a^	2.90 × 10^−4^	0.035
GH44	cellulase family J	1.80 × 10^−5^	5.86 × 10^−5^	2.28 × 10^−5^	1.00 × 10^−5^	0.344
GH45	cellulase family K	6.21 × 10^−4 b^	4.38 × 10^−4 b^	2.57 × 10^−3 a^	3.00 × 10^−4^	<0.001
GH48	cellulase family L	3.25 × 10^−3a^	1.31 × 10^−3 b^	2.64 × 10^−3 a^	2.70 × 10^−4^	<0.001
GH5	cellulase family A	1.12 × 10^−2 b^	1.53 × 10^−2 b^	2.44 × 10^−2 a^	1.96 × 10^−3^	0.003
GH6	cellulase family B	1.01 × 10^−4^	2.98 × 10^−4^	1.46 × 10^−4^	5.00 × 10^−5^	0.195
GH8	cellulase family D	2.04 × 10^−4^	2.06 × 10^−4^	2.35 × 10^−4^	3.00 × 10^−5^	0.917
GH9	cellulase family E.	1.21 × 10^−4 b^	9.70 × 10^−4 a^	1.04 × 10^−4 b^	1.70 × 10^−4^	0.049
GT2	cellulose synthase	1.40 × 10^−3 a^	1.15 × 10^−3 a,b^	8.28 × 10^−4 b^	1.00 × 10^−4^	0.044
Total		7.16 × 10^−2 b^	5.59 × 10^−2 c^	8.54 × 10^−2 a^	3.94 × 10^−3^	<0.001

^a–c^ means within a row with different letters differed significantly (*p* < 0.05); SEM = standard error of the mean. CON = control diet, HC = high-concentrate diet, HCT = high-concentrate diet supplemented with 180 mg thiamine/kg DM, CBM = Carbohydrate-binding modules, GH = Glycoside hydrolase, GT = Glycosyl transferase.

**Table 3:** Effects of high-concentrate diet and thiamine supplementation treatment on relative abundances of genes encoding starch-degrading enzymes.

Enzyme	Functions	CON	HC	HCT	SEM	*p*-Value
CBM20	granular starch-binding	3.43 × 10^−3 a^	1.90 × 10^−3 b^	2.43 × 10^−3 b^	2.300 × 10^−4^	0.008
CBM25	starch-binding	9.03 × 10^−3 b^	8.30 × 10^−3 b^	1.49 × 10^−2 a^	1.060 × 10^−3^	0.004
CBM26	starch-binding	1.38 × 10^−3 b^	2.38 × 10^−3 a,b^	3.07 × 10^−3 a^	2.900 × 10^−4^	0.035
CBM34	granular starch-binding	1.80 × 10^−5^	5.86 × 10^−5^	2.28 × 10^−5^	1.000 × 10^−5^	0.344
GH13	α-amylase	3.25 × 10^−3 a^	1.31 × 10^−3 b^	2.64 × 10^−3 a^	2.700 × 10^−4^	<0.001
GH15	glucoamylase	1.12 × 10^−2 b^	1.53 × 10^−2 b^	2.44 × 10^−2 a^	1.960 × 10^−3^	0.003
GH31	α-glucosidase	3.63 × 10^−4^	4.11 × 10^−4^	1.86 × 10^−4^	5.000 × 10^−5^	0.176
GH4	β-amylase	2.58 × 10^−3 a^	3.33 × 10^−4 b^	1.69 × 10^−3 a^	3.300× 10^−4^	0.003
GH57	α-amylase and amylopullulanase	1.01 × 10^−4^	2.98 × 10^−4^	1.46 × 10^−4^	5.000 × 10^−5^	0.195
GH63	α-glucosidase	5.16 × 10^−3 a^	3.13 × 10^−3 b^	3.75 × 10^−3 b^	3.300 × 10^−4^	0.015
GH77	amylomaltase	2.04 × 10^−4^	2.06 × 10^−4^	2.35 × 10^−4^	3.000 × 10^−5^	0.917
GH97	glucoamylase	1.21 × 10^−4 b^	9.70 × 10^−4 a^	1.04 × 10^−4 b^	1.700 × 10^−4^	0.049
GH119	α-amylase	6.21 × 10^−4 b^	4.38 × 10^−4 b^	2.57 × 10^−3 a^	3.00 × 10^−4^	<0.001
GT35	glycogen or starch phosphorylase	1.40 × 10^−3 a^	1.15 × 10^−3 a,b^	8.28 × 10^−4 b^	1.000 × 10^−4^	0.044
GT5	starch glucosyltransferase	2.80 × 10^−4^	8.59 × 10^−4^	4.90 × 10^−4^	2.200 × 10^−4^	0.180
Total		2.33 × 10^−2 b^	2.29 × 10^−2 b^	3.36 × 10^−2 a^	2.560 × 10^−3^	0.016

^a,b^ means within a row with different letters differed significantly (*p* < 0.05); SEM = standard error of the mean. CON = control diet, HC = high-concentrate diet, HCT = high-concentrate diet supplemented with 180 mg thiamine/kg DM, CBM = Carbohydrate-binding modules, GH = Glycoside hydrolase, GT = Glycosyl transferase.

## Supplementary Materials

The following are available online at https://www.mdpi.com/2076-2615/10/2/304/s1, Table S1: Ingredients and chemical composition of the experimental diets, Table S2: Effects of high-concentrate feeding and thiamine supplementation on dry matter intake, milk production, ruminal pH, ruminal thiamine, and VFAs content, Table S3: Quantitative information and quality control of sequencing, Excel S1: CAZymes identified in individual dairy cows.
