# Supplementary material for: Metagenomic Insights into Effects of Thiamine Supplementation on Carbohydrate-Active Enzymes’ Profile in Dairy Cows Fed High-Concentrate Diets
Source: Animals (Basel). 2020 Feb 14;10(2):304. doi: 10.3390/ani10020304 (PMC7070242; doi:10.3390/ani10020304)
Supplement: Supplementary file 1 [file animals-10-00304-s001.zip › Supplementary Table S1.docx]

Table S1. Ingredients and chemical composition of the experimental diets

| Items | Control diet | High-contrate diet |
| --- | --- | --- |
| *Ingredients( % of DM)* |  |  |
| Chinese wildrye | 11.0 | 5.0 |
| Corn silage | 34.0 | 20.0 |
| Alfalfa hay | 15.0 | 15.0 |
| Ground corn | 10.0 | 30.0 |
| Soybean meal, 43% CP | 14.0 | 14.0 |
| Cottonseed meal | 5.0 | 5.0 |
| Distillers dried grains with solubles | 5.0 | 5.0 |
| Whole cottonseed | 3.0 | 3.0 |
| Limestone meal | 1.0 | 1.0 |
| Calcium hydrogen phosphate | 0.7 | 0.7 |
| Sodium chloride | 0.5 | 0.5 |
| Premix^1^ | 0.8 | 0.8 |
| *Nutrient composition (% of DM)* | | |
| NE_L_^2^, Mcal/kg | 1.58 | 1.68 |
| CP | 18.16 | 18.10 |
| Starch | 19.95 | 30.82 |
| NDF | 36.18 | 27.61 |
| ADF | 23.43 | 17.72 |
| NFC^3^ | 32.67 | 45.74 |
| Ether extract | 4.61 | 4.20 |
| Ash | 5.04 | 4.35 |
| Calcium | 0.88 | 0.84 |
| Phosphorus | 0.55 | 0.55 |

^1^Premix contained (per kg): 2142 mg of Cu (as sulfate); 15428 mg of Mn (as sulfate); 15428 mg of Zn (as sulfate); 28 mg of Co (as chloride); 231 mg of I (as iodate); 57 mg of Se (as selenite); 2285000 IU of vitamin A; 457000 IU of vitamin D;11400 mg of vitamin E.

^2^NE_L_ was estimated according to NRC (2001).

^3^NFC = 100 – (% NDF + % CP + % ether extract + % ash) (NRC, 2001)
