# Supplementary material for: Metagenomic Insights into Effects of Thiamine Supplementation on Carbohydrate-Active Enzymes’ Profile in Dairy Cows Fed High-Concentrate Diets
Source: Animals (Basel). 2020 Feb 14;10(2):304. doi: 10.3390/ani10020304 (PMC7070242; doi:10.3390/ani10020304)
Supplement: Supplementary file 1 [file animals-10-00304-s001.zip › Supplementary Table S2.docx]

Table S2. Effects of high-concentrate feeding and thiamine supplementation on dry matter intake (DMI), milk production, ruminal pH, ruminal thiamine and VFAs content

| Item | Experimental Treatments | | | SEM | *P*-value |
| --- | --- | --- | --- | --- | --- |
|  | CON | HC | HCT |  |  |
| DMI (kg/d) | 21.68^a^ | 19.07^c^ | 20.78^b^ | 0.278 | 0.014 |
| Milk production (kg/d) | 27.28^a^ | 22.12^c^ | 23.28^b^ | 1.894 | 0.001 |
| Milk fat (%) | 3.85^a^ | 3.39^b^ | 3.68^a^ | 0.126 | 0.046 |
| Milk protein (%) | 3.11 | 3.05 | 3.08 | 0.061 | 0.106 |
| Ruminal pH | 6.45^a^ | 5.58^c^ | 6.12^b^ | 0.194 | 0.016 |
| Ruminal Thiamine (ug/L) | 16.16^a^ | 9.51^c^ | 13.53^b^ | 1.933 | <0.001 |
| Acetate (mmol/L) | 43.24^a^ | 42.62^b^ | 44.07^a^ | 1.273 | 0.038 |
| Propionate (mmol/L) | 12.89^b^ | 13.85^a^ | 11.84^c^ | 0.632 | 0.027 |
| Butyrate (mmol/L) | 10.77 | 10.35 | 10.82 | 0.137 | 0.356 |
| Valerate(mmol/L) | 1.77^b^ | 2.14^a^ | 1.96^ab^ | 0.064 | 0.041 |
| Isovalerate (mmol/L) | 2.03^b^ | 2.34^a^ | 1.99^b^ | 0.060 | 0.016 |
| TVFA (mmol/L) | 70.32 | 70.98 | 70.48 | 1.836 | 0.156 |

^1^a,b,c means within a row with different letters differed significantly (P < 0.05); SEM, standard error of the mean.

^2^CON = control diet; HC = high-concentrate diet); HCT = high-concentrate diet supplemented with thiamine; TVFA = total volatile fatty acid.
