# Supplementary material for: Metagenomic Insights into Effects of Thiamine Supplementation on Carbohydrate-Active Enzymes’ Profile in Dairy Cows Fed High-Concentrate Diets
Source: Animals (Basel). 2020 Feb 14;10(2):304. doi: 10.3390/ani10020304 (PMC7070242; doi:10.3390/ani10020304)
Supplement: Supplementary file 1 [file animals-10-00304-s001.zip › Supplementary Table S3.docx]

Table S3. Quantitative information and quality control of sequencing

| Sample | | Total Reads | Average Read Length (bp) | Contigs | Predicted Genes | N50 | N90 | | GC content |
| --- | --- | --- | --- | --- | --- | --- | --- | --- | --- |
| CON1 | 38117710 | | 144.14 | 136750 | 243399 | 873 | | 402 | 49.73% |
| CON2 | 46684704 | | 142.97 | 150605 | 261343 | 855 | | 393 | 49.02% |
| CON3 | 60005524 | | 144.36 | 205536 | 355099 | 837 | | 387 | 48.87% |
| CON4 | 49418758 | | 144.54 | 185420 | 305814 | 882 | | 426 | 51.60% |
| HC1 | 41149472 | | 142.33 | 130666 | 258388 | 891 | | 369 | 50.30% |
| HC2 | 45783576 | | 144.8 | 164161 | 360929 | 762 | | 297 | 46.59% |
| HC3 | 66780112 | | 144.91 | 266675 | 539128 | 813 | | 327 | 47.17% |
| HC4 | 38925364 | | 144.97 | 140820 | 280129 | 783 | | 321 | 47.10% |
| HCT1 | 51513440 | | 144.22 | 177350 | 270252 | 897 | | 447 | 41.29% |
| HCT2 | 56614214 | | 143.39 | 180487 | 283991 | 879 | | 423 | 43.58% |
| HCT3 | 44973212 | | 144.28 | 161734 | 254637 | 861 | | 402 | 41.59% |
| HCT4 | 38932060 | | 143.7 | 132493 | 180726 | 867 | | 456 | 36.36% |

^1^CON = control diet; HC = high-concentrate diet; HCT = high-concentrate diet supplemented with thiamine
